# Fourth International Dental Congress, St. Louis, Mo., August 29 to September 3, 1904

**Published:** 1904-10

**Authors:** 


### FOURTH INTERNATIONAL DENTAL CONGRESS, ST. LOUIS, MO., AUGUST 29 TO SEPTEMBER 3, 1904.

*Opening Session—Monday, August 29, 1904.*

THE first general session was called to order at 11 A.M. Monday, August 29, by Hon. Howard J. Rogers, Director of Congresses of the Universal Exposition.

Rev. H. H. Gregg, St. Louis, invoked divine blessings on the deliberations of the Congress.

Hon. Howard J. Rogers, in opening the Congress, and welcoming the members on behalf of the Universal Exposition, spoke as follows:

MEMBERS OF THE FOURTH INTERNATIONAL DENTAL CONGRESS, LADIES, AND GENTLEMEN,—It gives me great pleasure to welcome you to this city to-day. To the members of the Fourth International Dental Congress; to the members of the National Dental Association; to the foreign delegates who are accredited by their respective governments; to the members of the profession, whether in this country or abroad, the Exposition extends a most cordial welcome, because we are glad to have you with us.

The profession of dentistry has made enormous strides during the past ten years, and our States are busily engaged at the present time in passing laws which shall govern the entrance to the study of this profession, and which shall improve the curriculum and govern the entrance to the profession itself. We expect, therefore, that from your sessions this week much of good and much of profit will accrue to this and the various States connected with your profession, and we expect from a legislative stand-point to receive valuable advice in framing proper laws for the development of dentistry. We hope, however, from an exposition standpoint, that you will find time during your deliberations to visit us at the great Exposition in the western part of the city, which should appeal to you sympathetically because it has been promoted upon educational lines. Our appeal to the government for funds, to foreign governments for co-operation, to the States of the Union for support, has been based upon this plea of education,—of course, meant in its broadest sense,—the education which comes to the people from observing arts and architecture, from exhibits which we have grouped together with due regard to their interrelation and interdependence, whereby the raw material is taken and under the eyes transposed into the finished article ready for the markets of the world. These exhibits have all been prepared with great expense and care, and we hope that you will avail yourselves of the opportunities there presented. If you are seeking for mental development this week, you will find it, I am sure, in the fifteen large buildings, crowded with the best things from all parts of the world, and from the many State and country exhibits surrounding them. If you are in search of physical development, you will find it, I am sure, while roaming over the twelve hundred acres comprised in the Exposition grounds. If you are seeking for relaxation and rest, we have to commend you to that apparatus of amusement, the Pike. All this we extend to you.

A very material part of the development of this Exposition has been a series of congresses and conventions. We have had something like two hundred conventions held under the Exposition auspices. To these we have given every opportunity and attention to make it a satisfactory meeting-place. We have scheduled twenty international congresses connected with the different sciences and professions, and these will culminate in the latter part of next month in the great Congress of Arts and Sciences which can be designated as a symposium of the scientific work of the world.

The Fourth International Dental Congress is the third in number of this great series of international congresses, and perhaps the greatest of any preceding it. In the organization of these various congresses it has been our policy to work through the national association of this country allied with that specialty, believing that it was best to do so because it would bring the people interested in that science or in that profession closely in touch with its development, and furthermore because we believed that the best interests of the Exposition and of the profession concerned would be promoted simultaneously. For that reason we have worked in your own case through the National Dental Association, and made the committee appointed by that Association our Committee of Organization, and we have given them every support, believing that they represented the best element of the profession in all parts of the country, or else they would not have been certified to by the National Association.

No one who has not had experience in the development and promotion of a great international congress can have any idea of the amount of work, the amount of time, and the amount of sacrifice necessary to bring it to a consummation. I venture to say that if every member of the Committee of Organization could have known two years ago what they know to-day of what was required of them in work, money, and effort, they would all have been very reluctant to accept the commission. It means a great deal of hard work and much criticism. Every committee of organization, however, must have two things fixed before them in order to bring about a successful congress. They must have a programme which in strength and timeliness of subject-matters should be of special interest to its profession. It must also bring together an audience capable of appreciating that programme. These two objects must be sought, and I would be remiss of my duty and profligate of my opportunities if I did not take this occasion on the part of the Exposition to render thanks to the Committee of Organization for the magnificent work they have done since they were appointed. To the chairman, who has looked particularly after the funds and the many and varied details of organization, all of which have been conducted with consummate executive ability; to the secretary, who has had a correspondence which runs into reams and reams of paper; to every member of the Committee of Organization, who has done well and to the best of his ability the particular duty ascribed to him,—again on the part of the Exposition I express our appreciation of their work.

We come now, gentlemen, to the permanent organization of the Fourth International Dental Congress. Following the procedure which has obtained in all of the other international congresses held here in our city under our auspices, some time ago we instructed the Committee of Organization to make a permanent roster of officers to preside over this convention, believing that by so doing we were contributing to the stability of the Congress, to the contentment of individuals, and to the permanence of the programme, and that we were working for the best interests of the Congress. We have found, however, that there is a strong opinion among the members of this convention—whose opinion we most highly respect—that the report of the nominating committee of the Committee of Organization should be submitted to your honorable body for ratification (applause), and at the special request of the chairman of the Committee of Organization, Dr. Burkhart, and at the request, passed in due form, of the committee, we will now rescind that former order, and present to this convention the nominations individually, so that there may be an expression of opinion as to the gentlemen who shall preside over you during the Congress.

The question therefore is, What action shall be taken upon the original report of the nominating committee, certified to us in due form by the Committee of Organization, nominating Dr. H. J. Burkhart for President of the Fourth International Dental Congress?

*Dr. B. Holly Smith,* Baltimore, Md.—Mr. Director, it is more than pleasant for those who have been interested in the organization of this Congress to have your public commendation of the work of the Organization Committee.

Townsend, in a little book entitled "The Art of Speech," quotes from an unknown writer the following: "The ball of discord has been thrown in our midst, and unless it be nipped in the bud it will burst into a conflagration that will deluge the world." Mr. Director, I cite this quotation because it is my personal desire that you should not regard these evidences of contention as a serious matter. It may be that some simple men in this audience may have a ball or two in their pockets, but this ball, if it exist, is not of a kind with which to start a fire, nor, if there should be some little balls of feeling, is there any probability that they will deluge the world and interfere with the work of this Congress. I have personally known some of the members of this committee for a number of years, the chairman especially, before he became a practitioner of dentistry. I have known him to be an honorable, upright, conscientious, energetic man in all of his professional work, and I feel that this Congress will do itself credit, and do the graceful thing in honoring him who has borne the brunt of the work of organizing this Congress; and I therefore move you, Mr. Director, that the action of the nominating committee in nominating him for the presidency of this Congress be confirmed, and that Dr. H. J. Burkhart be elected President. (Applause.)

*Dr. R. H. Hofheinz,* Rochester, N. Y.—Mr. Director, as a member of the Committee of Organization, of which you have spoken so flatteringly and appreciatively, I take great pleasure in seconding the motion of Dr. Smith that the action of the nominating committee be confirmed, and that Dr. Burkhart be elected the President of the Fourth International Dental Congress.

*Mr. Rogers.*—The question before the house is, Shall the action of the nominating committee for the presidency of this Congress, as recommended by the Committee of Organization, be ratified?

The motion was carried, and Mr. Rogers declared the action of the nominating committee in nominating Dr. Burkhart for President ratified and adopted.

*Dr. C. N. Johnson,* Chicago.—Mr. Director, I should like to move that this motion be made unanimous.

Mr. Rogers put the motion, and the election of Dr. Burkhart for President was made unanimous.

Dr. Burkhart was escorted to the platform, and Mr. Rogers introduced him as follows: "Gentlemen of the convention, I have the honor to introduce to you your presiding officer, Dr. H. J. Burkhart." (Applause.)

*Dr. Burkhart.*—Gentlemen of the Fourth International Dental Congress, I thank you deeply for this expression of confidence, and for confirming the action of the nominating committee and the work which that committee has tried to do conscientiously and for the best interests of this Congress.

The next order of business will be the election of the Secretary-General.

*Dr. James Truman,* Philadelphia, Pa.—Mr. President, I wish to nominate for the office of Secretary-General Dr. Edward C. Kirk, of Philadelphia. (Applause.) I take great pleasure in saying that Dr. Kirk has from the very inception of this movement done a large portion of the work connected with it. By day and by night that gentleman has worked faithfully up to the present moment, and he resigned from the Secretary's work simply because be found that the committee had decided to elect the officers of the Congress permanently, and because of the fact that his word, which had been sent broadcast over the world, was pledged to the principle that the Congress would elect its own officers. He had said that the Congress would and should elect its own officers, and therefore he would not allow his name to be used when the time for electing officers came. Now, the question before you is whether you will elect this man, who has done so much for this Congress, or elect some one else. I have no objection to that some one else, but I do object to allowing an individual who has accomplished so much to be overlooked by the Congress. I do not care personally who the officers of this Congress are; that is not my object—my desire is for the advancement of the dental profession scientifically; but I want you to understand that when a man has done all that Dr. Kirk has done for this Congress, it is our right and our duty to elect him, and I therefore nominate him for Secretary-General of the Fourth International Dental Congress. (Applause.)

*Dr. E. T. Darby,* Philadelphia, Pa.—Mr. President, it gives me great pleasure to second the nomination which has been made for Dr. Kirk as the Secretary-General of the Fourth International Dental Congress. (Applause.)

*Dr. G. V. I. Brown,* Milwaukee, Wis.—Mr. President, the motion is out of order. The method of procedure begun by Mr. Rogers was that each of these nominations be submitted to the Congress for ratification or non-ratification of the action of the Committee of Organization.

*Dr. William Conrad,* St. Louis, Mo.—As a member of the committee which nominated the officers for the Fourth International Dental Congress I would state that we nominated Dr. Burkhart for President, Dr. Kirk for Secretary-General, and Dr. Finley for Treasurer. Dr. Kirk resigned his position after being elected, and Dr. A. W. Harlan was elected to take his place. I move, Mr. President, that this Congress indorse the action of the nominating committee in nominating Dr. A. W. Harlan for Secretary-General.

The motion was seconded by Dr. J. Y. Crawford, Nashville, Tenn.

Dr. Conrad's motion was lost.

*Dr. Truman.*—Mr. President, I now renew my nomination of Dr. E. C. Kirk for Secretary-General of the Congress.

*Dr. William Conrad,* St. Louis.—I nominate Dr. A. W. Harlan for Secretary-General of the Fourth International Congress.

A rising vote was taken on the two nominees, and resulted in the election of Dr. Kirk.

Dr. E. C. Kirk was escorted to the stage, and was presented to the audience by the President.

*Dr. E. C. Kirk.*—My friends, I have neither the strength nor nervous energy to at this moment say more than that I thank you from the bottom of my heart for two things: First, for all that this means of personal regard and confidence; and second, that it represents, I believe, the allegiance to the principle which has been so gracefully conceded by the Committee of Organization,—namely, the right of every man to express his views upon this question. (Applause.)

*The President.*—The next order of business is the ratification of the action of the nominating committee in nominating Dr. M. F. Finley for Treasurer of the Congress.

*Dr. William Conrad,* St. Louis, Mo.—Mr. President, I move that the nomination of Dr. Finley for Treasurer be ratified by this body.

The motion was put before the house, and Dr. Finley was duly elected Treasurer of the Fourth International Dental Congress.

Dr. Finley was escorted to the platform and introduced by the President as follows: "Gentlemen of the Fourth International Dental Congress, I have great pleasure in presenting to you the gentleman who presides over the funds of this Congress." (Applause.)

*Dr. Finley.*—Mr. President and gentlemen, I would like to be able to thank you as gracefully as has Dr. Kirk, and if you will accept his words for mine, I will thank you.

The President next introduced Dr. C. S. Butler, Buffalo, N. Y., chairman of the Finance Committee, who thanked the members for their hearty co-operation in carrying out the work of his committee.

The President then invited all of the honorary presidents and vice-presidents in the audience to come upon the platform.

*The President.*—Gentlemen and ladies of the convention, I have pleasure in presenting to you my long-time friend and colleague, Dr. C. N. Johnson, of Chicago.

*Dr. Johnson.*—Mr. President and gentlemen of the Congress, this is entirely a surprise to me, but I do want to thank most heartily your presiding officer for giving me the pleasure of looking you in the face, and begging you to go to work from this time forward with the one object of making this the best meeting ever held in the history of dentistry. If perchance there has been any feeling developed, let us crush it out and as one man let us all work together to do the best we can for the profession of dentistry.

*The President.*—The governor of Missouri was invited to make an address of welcome on this occasion, but was unable to be here, and in his absence you will listen to a letter from him, which will be read by the Secretary-General.

Dr. Kirk, the Secretary-General, then read the following communication from Governor Dockery:

St. Louis, Mo., August 28, 1904.

"To Dr. B. L. Thorpe:

My dear Sir,—I profoundly regret my inability to be present tomorrow and welcome the delegates to the Fourth International Dental Congress. It would have been a real pleasure for me, in behalf of the people of Missouri, to have extended a cordial greeting to this large body of representative and intelligent citizens. Please present to the Congress my regrets that official duties prevent my attendance, and also convey my best wishes for a pleasant and instructive occasion.

"Your friend,

"A. M. Dockery."

*The President.*—Ladies and gentlemen, I have the honor of presenting to you the honored president of the National Dental Association, Dr. C. C. Chittenden, of Madison, Wis., who will now address you.

*Dr. Chittenden.*—Mr. President, ladies, and gentlemen, great undertakings can only be brought to a successful issue by a careful, well-planned course of procedure and development, controlled by an intelligent comprehension of the objects sought to be attained. Time, patience, and unswerving courage and fidelity—regardless of all personal sacrifice—must be freely thrown into the balance by those who undertake to create and set in motion the machinery required to successfully bring together representative men from all the civilized nations of the earth for the uplifting of a common cause.

Two years ago the National Dental Association, which body I have the honor to officially represent to-day, received an invitation from the officials of the Louisiana Purchase Exposition to create and put in operation an International Dental Congress, to be held in St. Louis. That invitation was accepted, and a commission of fifteen was appointed to the arduous undertaking. The work was taken up by that commission, and despite the thousand difficulties and obstacles that beset the way,—some of them apparently impregnable as Gibraltar itself,—the result of its labors stands before us to-day in the form of a noble, completed, symmetrical achievement, —the Fourth International Dental Congress.

Too much cannot be said in appreciation of the untiring labor and self-sacrifice through which this result has been brought about. On behalf of the National Dental Association I here publicly thank the commission of fifteen collectively and individually for their faithful performance of the duties set them.

This Congress, whose opening we are met to celebrate, could not have been made possible but for the never-failing guidance, counsel, and (when needed) authoritative direction of a master mind in the person of the Director of Congresses, Mr. Howard J. Rogers, to whom each one of us here owes a deep debt of gratitude.

The deep interest created in this enterprise throughout the countries of the world is clearly evidenced by the presence here to-day of eminent, illustrious representatives and large delegations of dentists from practically every civilized nation of the earth. On behalf of the National Dental Association I extend to these professional brothers gathered under this roof from the very ends of the earth, seeking the development of scientific light and knowledge in their chosen calling,—to all these I extend the hand-clasp of greeting and warmest words of welcome.

Under the skilled business management of the Finance Committee there is every reason to expect that this Congress will present a happy contrast, as to its final solvency, to most such undertakings. An ample equipment for each of the sections has been assured.

Finally, to all who have in any way contributed to the success of this great enterprise I here offer the sincere thanks of the National Dental Association. (Applause.)

*The President.*—The address of welcome on behalf of the State of Missouri will be delivered by the gentleman to whom I believe belongs the credit of discovering this Congress. I take great pleasure in introducing to you Dr. Burton Lee Thorpe, of St. Louis, Mo. (Applause.)

ADDRESS OF WELCOME ON BEHALF OF THE STATE OF MISSOURI.

MR. PRESIDENT, AND MEMBERS OF THE FOURTH INTERNATIONAL DENTAL CONGRESS,—Proud to be an American citizen, prouder still to be an American dentist, and to have aided in a humble way in making it possible that this great meeting is held, this is the happiest opportunity of my professional career,—to be delegated the very delightful duty of bidding you a most cordial and sincere welcome on behalf of the profession of the State of Missouri and the city of St. Louis.

Recognizing that each of you delegates coming from foreign countries and the States, Territories, and provinces of North America represent the best interests of the profession of your various localities, and that each of you has contributed either to the science, art, or literature of dentistry, I am frank to confess that the profession of Missouri feels highly honored to greet and welcome such a distinguished body of representative men and women of the dental profession.

It is fitting, indeed, that during the great Universal Exposition now in progress at the western portals of our city, that is held in commemoration of that historic event when Napoleon Bonaparte, with a stroke of the pen, signed away and gave to Thomas Jefferson and the American people an empire of which imperial Missouri is the most important State,—it is fitting, I say, at this time that there be held, as one of a series of many important educational and scientific congresses, the Fourth International Dental Congress.

It is the spirit of the Exposition to show the achievement of human progress, so aptly illustrated by Mr. Chang You Tong, the talented interpreter of China of this Congress, in the following sentiment:

> "Well blessed with man's success, Columbia fêtes
> In festal hall the nations and her States;
> From every clime her honored guests have come,
> From far Cathay and Europe's Christendom,
> From silver-mantled regions of the poles,
> From golden shores where sparkling river rolls;
> Her States, like jewels in a coronet,
> Around the hall her loving hands have set.
> The wine of friendship bubbles in the glass,
> From hand to hand the fragrant roses pass;
> The song of thanks is swelled by every tongue,
> The nation's hymn is sung by old and young.
> Rejoice, Columbia! Lead the gallant van,
> And show the world the worth of man as man:
> Be thou the champion of the human race,
> And break the chains which manhood may disgrace.
> The nations, young and old, to thee resign
> The sacred sceptre and a task divine."

It is also the spirit of this Congress to welcome our professional *confrères* from every civilized country, and to unite with them in comparing the wonderful strides the humane and beneficent profession of dentistry has made by enmassing the investigators, teachers, journalists, and active progressive practitioners of dental surgery, the recognized leaders in art, science, and technique, to demonstrate both clinically and theoretically the latest and best in odontological science.

We are gratified that in the master minds of two American dental pioneers originated the three most potent factors in dental education,—the organization of the first dental college, the first dental journal, and the first dental society that brought our calling out of chaos, and made it blossom from a trade into a profession. We know that yesterday's achievements are not forgotten in the onward movement of to-day, when all nations in reverential homage do honor to the memory of the progenitors of dental science,—Horace H. Hayden and Chapin A. Harris.

We are not unmindful, however, of the achievements of the stanch pioneers of Europe, to whom we also must give ample credit for their contributions to our profession.

One hundred years ago, when St. Louis was a frontier trading-post on the banks of yonder river, records say that the first regular dental practitioner was Dr. Paul, a Frenchman of New Orleans, who located here in 1809, soon to be followed by other pioneers, such as Isaiah Forbes, B. B. Brown, Edward Hale, Sr., A. M. Leslie, Isaac Comstock, Aaron Blake, J. S. Clark, H. E. Peebles, C. W. Spaulding, Henry Barron, Homer Judd, C. W. Rivers, Henry S. Chase, William H. Eames, Edgar Park, William N. Morrison, and lastly our distinguished Henry J. McKellops, whose names will ever illumine the pages of Missouri dental history.

This coterie of men stood for progress, and were foremost in city, State, and national dental affairs, and I thank God that the better element of their successors are also imbued with the same progressive spirit, which is proved by their loyalty and liberality in making this Congress a success. On behalf of these self-sacrificing, broad-minded, liberal-spirited dentists of St. Louis and Missouri, I welcome you!

We welcome all nations. We welcome you of Spain, whose generous Queen Isabella long ago equipped the fleet that brought Columbus on his voyage of discovery to our shores. Our hearts also go out to you for the courteous and chivalrous treatment tendered our sailors while the captives of your gallant Admiral Cervera at Santiago.

To you, sons of England, the mother of America: We bid you welcome! Once we were your unruly child; you tried to chastise us, but we had outgrown our swaddling-clothes—you failed; but now the British lion and the American eagle are bosom companions. We heartily appreciate the recognition England has granted that distinguished scientist, our fellow-countryman, J. Leon Williams.

To Sweden, who gave us Ericsson, and to Norway, who gave us so many of your sturdy sons and fair daughters who have by their industry made parts of America blossom as a garden: We bid you welcome!

To France, who in the dark days of the American Revolution sent us the gallant Marquis de Lafayette and Count Rochambeau and their thousands of fellow-patriots who did so much to aid us to gain our independence, we are especially indebted. With these generals of army and navy came two men who have left their imprint indelibly stamped on American dentistry,—Joseph Françoise Lemaire and James Gardette, distinguished as skilled operators, contributors to dental literature, and the pioneers in introducing dental surgery in America: We welcome you! Our national emblem, symbolic of free thought and free speech, is the Stars and Stripes. I believe if the American people were asked to add to it, in recognition of the brotherly love between France and America they would unanimously choose the *fleur-de-lis*.

To Germany, whose countrymen have done so much in developing America and have made such excellent citizens: We bid you welcome! and we thank you for the fraternal recognition you have given those two distinguished American practitioners who are the foremost in the world in their specialties,—W. D. Miller and N. S. Jenkins.

Lord Bacon says, "Every man owes a debt to his profession," and we feel that you who speak in different tongues, who have crossed the seas to attend this Congress, have at least in part liquidated your indebtedness to the profession.

To all nations: to all members of the Fourth International Dental Congress: We bow our heads in salute to you; we bid you welcome from the bottom of our hearts! Our homes are open to you, our hands are at your service. We recognize the fact that you represent the intellect and flower of dentistry of the world, and the profession of Missouri and of St. Louis are flattered to have you here. It is our hope that you may reap untold benefit from this historic meeting, and when you return to your respective homes, may you carry pleasant memories of our people, State, and city; and when your life's work is ended, may you each realize the reward that Kipling so beautifully pictures as the heaven of the honest workingman as epitomized by the painter:

"When earth's last picture is painted and the tubes are twisted and dried,
When the brightest colors have faded, and the youngest critic has died,
We shall rest, and faith! we shall need it—lie down for an æon or two,
Till the Master of all good workmen shall put us to work anew.
And those who were good shall be happy. They shall sit in a golden chair;
They shall splash at ten-league canvas with brushes of comet's hair;
They shall find real saints to draw from,—Magdalene, Peter, and Paul;
They shall work for an age at a sitting, and never be tired at all!
And only the Master shall praise us, and only the Master shall blame;
And no one shall work for money, and no one shall work for fame;
But each for the joy of working, and each in his separate star,
Shall draw the Thing-as-he-sees-it, for the God of Things-as-they-are!"

*The President.*—I now have pleasure in presenting to you the vice-president for America of the International Dental Federation, who will welcome the foreign delegates to the Fourth International Dental Congress.

Dr. A. W. Harlan, of New York, delivered the following address:

ADDRESS OF WELCOME TO THE FOREIGN DELEGATES.

MR. PRESIDENT, LADIES, AND GENTLEMEN, AND MEMBERS OF THE CONGRESS FROM FOREIGN COUNTRIES,—I am indeed fortunate to be able this day to greet you as the official mouth-piece of the Committee of Organization of this Congress. It looked for a long time as though the Fédération Dentaire Internationale and the committee could not come together, but to-day all differences are settled, and we appear before you as a united profession.

The World's Dental Congresses have become fixed institutions in the same way that other congresses have been evolved. The profession needed congresses. The world would be benefited in consequence of their evolution, and we have them. They are a part of our armament, just as physiology and chemistry are a part of the education of the dentist.

The members of our profession in the United States having chosen me to extend to every one present from a foreign shore the right hand of fellowship, I give you a cordial fraternal welcome in this noble hall and in the presence of this multitude of our own people.

This is the second time in the history of our profession that an international dental congress has been convened in the United States,—1893 and 1904. The origin of dental congresses, as you well know, was in the land of Ambroise Paré, Roux, Fauchard, Jourdain, Lefoulon, Robin, Magitot, Paul Dubois, Lecaudey, and Godon. It is our hope and wish that every one here this day will feel that he is thrice welcome to participate in the programme of the week, and that all will feel that time and distance will count as nothing when they sum up the benefits derived from what they see and hear.

On behalf of the officers and the management of the Congress, and the united dental profession, I again bid you all a hearty welcome.

(To be continued.)

